# Advancing research and practice in HIV and rehabilitation: a framework of research priorities in HIV, disability and rehabilitation

**DOI:** 10.1186/s12879-014-0724-8

**Published:** 2014-12-31

**Authors:** Kelly K O’Brien, Francisco Ibáñez-Carrasco, Patricia Solomon, Richard Harding, Jessica Cattaneo, William Chegwidden, Jacqueline Gahagan, Larry Baxter, Catherine Worthington, Patriic Gayle, Brenda Merritt, Rosalind Baltzer-Turje, Nkem Iku, Elisse Zack

**Affiliations:** Department of Physical Therapy, University of Toronto, 500 University Avenue, Room 160, Toronto, ON Canada; Institute of Health Policy, Management and Evaluation, University of Toronto, 4th Floor, 155 College Street, Toronto, ON Canada; School of Rehabilitation Science, McMaster University, 1400 Main Street West, Room 403, Hamilton, ON Canada; Ontario HIV Treatment Network, 1300 Yonge Street, Suite 600, Toronto, ON Canada; Cicely Saunders Institute, School of Medicine, King’s College London, Bessemer Road, London, United Kingdom; AIDS Committee of Toronto, 399 Church Street, 4th Floor, Toronto, ON Canada; Barts and the London NHS Trust, London, United Kingdom; University College Hospitals NHS Foundation Trust, London, United Kingdom; School of Health and Human Performance, Dalhousie University, Stairs House, 6230 South Street, Halifax, NS Canada; Canadian Working Group on HIV and Rehabilitation, 600 Bay Street, Suite 600, Toronto, ON Canada; School of Public Health and Social Policy, University of Victoria, Human and Social Development Building, 3800 Finnerty Road, Victoria, BC Canada; Three Flying Piglets, London, United Kingdom; School of Occupational Therapy, Dalhousie University, Forrest Building, Room 215, 5869 University Avenue, Halifax, NS Canada; Dr. Peter AIDS Foundation, 1110 Comox Street, Vancouver, BC Canada

**Keywords:** HIV/AIDS, Rehabilitation, Disability, Research, Aging

## Abstract

**Background:**

HIV increasingly is experienced as a complex chronic illness where individuals are living longer with a range of physical, cognitive, mental and social health-related challenges associated with HIV, comorbidities and aging, a concept that may be termed ‘disability’. Rehabilitation such as physical therapy and occupational therapy can help address disability and has the potential to improve quality of life in people living with HIV. Hence, the role for rehabilitation in the context of HIV, aging and comorbidities is emerging. Our aim was to establish a framework of research priorities in HIV, disability and rehabilitation.

**Methods:**

We convened people living with HIV, clinicians, researchers, service providers, representatives from community-based organizations and policy and funding stakeholders to participate in the first International Forum on HIV and Rehabilitation Research. We conducted a multi-stakeholder consultation to identify current and emerging issues in HIV, disability and rehabilitation. Data were collated and analyzed using content analytical techniques.

**Results:**

Ninety-two participants attended the Forum from Canada, United Kingdom (UK), Ireland and the United States. Situated within three overarching themes (episodic health and disability across the life course; rehabilitation; and methodological advances), the Framework of Research Priorities in HIV, Disability and Rehabilitation includes six research priorities: 1) episodic health and disability; 2) aging with HIV across the life course; 3) concurrent health conditions; 4) access to rehabilitation and models of rehabilitation service provision; 5) effectiveness of rehabilitation interventions; and 6) enhancing outcome measurement in HIV and rehabilitation research. The Framework includes methodological considerations and environmental and personal contextual factors (or lenses) through which to approach research in the field. Knowledge translation should be implemented throughout the development and application of research knowledge to inform HIV clinical practice, programming and policy.

**Conclusions:**

These priorities highlight the emerging priorities of living long-term with HIV and outline a plan for HIV and rehabilitation research in resource-rich countries such as the UK and Canada.

**Electronic supplementary material:**

The online version of this article (doi:10.1186/s12879-014-0724-8) contains supplementary material, which is available to authorized users.

## Background

In developed countries such as Canada, and the United States, HIV is increasingly experienced as a chronic illness [1]. At the end of 2011, an estimated 71,300 people were living with HIV in Canada, and over a million in the United States; and at the end of 2012 an estimated 98,400 were living with HIV in the United Kingdom [2]-[4]. With advances in treatment, people with HIV are now aging and may approach life expectancy similar to uninfected populations [5]. For instance, by 2015, half the people living with HIV in the United States will be 50 years of age or older [6]. This trend may be similarly forecast in other developed countries for those with access to treatment.

Despite improvements in survival, people with HIV are living with a range of physical, cognitive, mental and social health-related challenges associated with HIV, comorbidities and aging, a concept that may be termed ‘disability’ [6]-[9]. A high prevalence of disability among people with HIV exists, with over 80% reportedly having experienced at least one impairment, activity limitation or social participation restriction [8]. Adults with HIV conceptualized disability as episodic, characterized by unpredictable periods of wellness and illness over time [10]. Episodes of disability may be exacerbated by extrinsic factors (stigma and lack of social support) and intrinsic contextual factors (aging, comorbidities) adding further complexity to the nature and extent of disability experienced by people living with HIV [11].

Rehabilitation in the context of HIV is defined as a dynamic process including any prevention and/or treatment activities and services that address body impairments, activity limitations and social participation restrictions for an individual [12]. Rehabilitation services such as physical therapy and occupational therapy can help address disability related to adverse effects of medications, fatigue, pain, cognitive problems, and issues related to employment; and has the potential to improve quality of life in people living with HIV [13],[14]. As the overall population ages, the demand for rehabilitation services is anticipated to increase, particularly for those living with HIV and other chronic and episodic illnesses [15],[16]. However, rehabilitation in the context of HIV is still emerging. Few rehabilitation professionals work in HIV care. A Canadian survey found that 61% of rehabilitation professional respondents had never knowingly worked with a person living with HIV and few HIV specialists refer their patients to rehabilitation [12],[17],[18]. Those living with HIV are often left having to utilize complementary and alternative medicines and therapies or community-based service organizations to address their health challenges [19]-[22]. Hence, there is a need for increased evidence to support and guide the role for rehabilitation in HIV clinical practice.

In 2008, the Canadian Working Group on HIV and Rehabilitation (CWGHR), a national, multi-sectoral organization whose aim is to improve the lives of people living with HIV through rehabilitation research, education and cross-sector partnerships, conducted a scoping study to identify research priorities related to HIV and rehabilitation [23],[24]. Since 2008, the following six research priorities have guided HIV and rehabilitation research and practice: 1) disability and episodic disability; 2) aging with HIV and concurrent health conditions; 3) HIV and the brain; 4) labour force and income support; 5) access to and effect of rehabilitation; and 6) development and evaluation of outcome measurement tools [23].

However, the field of HIV and rehabilitation has changed over the past five years. The rising prevalence of comorbidities including cardiovascular disease, diabetes [25], bone and joint disorders [26],[27], neurocognitive disorders [28], and non-AIDS-defining cancers [29] further add to the complexity of physical, mental and social disability experienced by adults with HIV over the life course [30]-[33]. A cross-sectional population based study in Ontario found that 34% of people with HIV were living with at least one other physical health condition and 39% a mental health condition. Prevalence of multi-morbidity, defined as combined physical and mental health comorbidity in addition to HIV, was 16% among participants; and this multi-morbidity increased with age [30]. Aging adults can additionally face challenges of ageism, stigma, mental health and lack of income and social support [34]-[36]. Research priorities have been developed in the context of HIV, aging and multi-morbidity [6], however they were not specific to the context of disability and rehabilitation. Hence, there was a need to establish research priorities in HIV and rehabilitation, to identify ongoing and new issues emerging in the field while framing them within the broader context of the clinical and research environment.

Our aim was to establish a framework of current and emerging research priorities in HIV, disability and rehabilitation from the perspective of people living with HIV, clinicians, researchers and representatives from community organizations. Identifying these priorities will help researchers, clinicians and members of the broader HIV community remain aware of the current disability and rehabilitation issues experienced by people living with HIV so that we may prioritize research efforts to establish best evidence to enhance overall HIV care, treatment and support.

## Methods

We conducted a series of consultations with people living with HIV, clinicians, academics, representatives from community-based organizations and policy and funding stakeholders who participated in the first International Forum on HIV and Rehabilitation Research. The goal of the Forum was to translate research evidence and to establish priorities in HIV and rehabilitation research. We reviewed the need for ethics approval with the University of Toronto, HIV/AIDS Research Ethics Board who confirmed that given the nature of our consultation was the form of a meeting proceeding, this work did not require ethics approval.

### Participants and procedure

We invited key stakeholders and broadly advertised the Forum in four countries (Canada, UK, Ireland, USA) to approximately 700 individuals with e-blasts, emails, websites, electronic newsletters, and posters through five collaborating organizations and one academic centre. This process was critical in order to mobilize diverse participants internationally in order to advance a community of learning and practice.

The two day Forum was held in Toronto, Ontario, Canada in June 2013 in collaboration with the Canada-United Kingdom HIV and Rehabilitation Research Collaborative (CUHRRC) and Canadian Working Group on HIV and Rehabilitation (CWGHR) [24],[37]. The event summarized the state of evidence in the field in the form of two plenary sessions and six Research Evidence Panel Sessions, each focused on one of the original research priorities. Each panel included two to five speakers who presented on research and program evaluation carried out in Canada, UK, Ireland, or the United States [38]. A combination of presentations and small and large group discussions provided opportunities to brainstorm new and emerging priorities. Participants discussed the implications of evidence presented for clinical practice, education, policy and research, as well as the cross-applicability of research and practice internationally [38]. The results of these discussions were reported synchronously by graduate student rapporteurs online [37].

### Data collection

Comments and recommendations related to research priority areas were documented through the following five methods: Prior to the Forum, 1) speakers were asked to submit responses to the following questions: ‘What are 2 new and emerging issues in the field of HIV, disability and rehabilitation?’ and ‘What are 2–3 key research priorities in the area of HIV, disability and rehabilitation essential for moving the field forward?’ During the Forum, 2) participants were asked to submit written responses to two questions similar to those above; 3) six graduate student rapporteurs documented the discussion throughout the Forum presentations, and large and small group sessions. 4) Participants were encouraged to document their ideas related to emerging research priorities as they pertained to each research evidence panel session and post them on a communal discussion board. At the end of the Forum, 5) participants were asked to complete an evaluation form that included the following item related to the research priorities: ‘In your opinion, what are 1 or 2 new and emerging issues that were not covered in the Forum?’ Also, we circulated a link to an online evaluation form one week after the Forum. Collectively, the comments, ideas and recommendations derived from these sources provided the foundation for identifying the new research priorities.

### Analysis

We collated and analyzed the data using content analytical techniques [39]. Two reviewers read through and independently coded the material using a line by line process. We developed a list of codes that were clustered into a Framework of current and emerging research priorities in HIV, disability and rehabilitation. Because the original six priorities provided the primary content of the Forum, emerging themes were informed by the original priorities. However, we removed any labels from data sources prior to coding that may have associated feedback with a specific panel session in order to remain open to the possibility for new priorities to emerge. The two reviewers (KKO and JC) met three times to discuss the content analysis and a team of six authors reviewed a preliminary version of the Framework for refinement.

## Results

The Forum was attended by 92 participants comprised of representatives from community-based organizations (including managers and directors) (24%; 21/88 participants who responded to this item), academic and community-based researchers (19%; 16/86), clinicians (16%; 14/86), research and program coordinators (12%; 10/86), people living with HIV and other chronic illnesses (11%; 9/86), educators (7%; 6/86), students (7%; 6/86), and other stakeholders including funders, consultants, policy stakeholders and media (7%; 6/86). Participants primarily worked in settings that included professional organizations that support service providers (24%; 21/88), universities (23%; 20/88), front-line community-based organizations (17%; 15/88), and organizations focused on research generation, funding, or translation (12%; 11/88). Most participants were from Canada (87%; n = 80), 10% (n = 9) were from the UK, 2% (n = 2) Ireland, and 1% (n = 1) from the United States. Given our recruitment strategy was primarily through collaborating organizations, participants were knowledgeable to the field of HIV and rehabilitation.

We received recommendations on research priorities from a combination of data sources including: 26 responses to the speaker and participant consultation; six rapporteur reports, 84 ideas posted on the discussion board after the research evidence panel sessions, and 22 responses to the item on the evaluation form.

### Framework of research priorities in HIV, disability and rehabilitation

**Figure 1:**
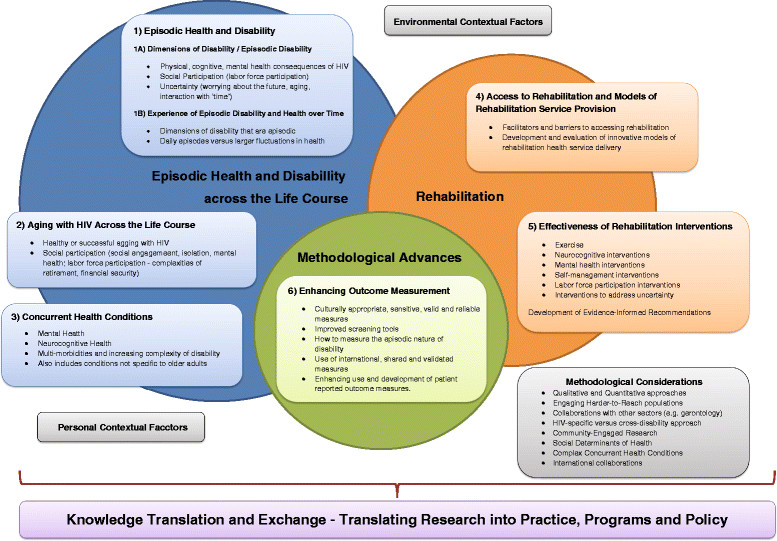
**Framework of research priorities in HIV, disability and rehabilitation.**

The Framework of Research Priorities in HIV, Disability and Rehabilitation reflects the increasing complexity of HIV associated comorbidity and the current changing environments that influence rehabilitation care delivery. The Framework includes six research priorities: 1) episodic health and disability; 2) aging with HIV across the life course; 3) concurrent health conditions; 4) access to rehabilitation and models of rehabilitation service provision; 5) effectiveness of rehabilitation interventions; and 6) enhancing outcome measurement in HIV and rehabilitation research (Figure 1). These priorities are situated within three broader content areas: A) episodic health and disability across the life course; B) rehabilitation; and C) methodological advances. The Framework includes methodological considerations and environmental and personal contextual factors (or lenses) through which to approach research in the field. Finally, knowledge translation should be implemented throughout the development and application of research knowledge generated from these priorities to inform clinical practice, programming and policy (Figure 1).

The Forum highlighted the important reciprocal relationship between HIV and rehabilitation research and clinical practice and policy. Specifically, clinical practice should inform research and newly generated research evidence should be translated to inform the future implementation of clinical practice, programs and policy [40],[41]. This Framework is both a research document and a knowledge, transfer and exchange (KTE) tool that may be used by researchers, clinicians, students, people living with HIV, and the broader HIV community as a foundation to inform future HIV, disability, and rehabilitation research. The priorities are in no particular order of importance.

### Content Area 1 - Episodic health and disability across the life course

The Forum highlighted the importance of increasing our understanding of living with HIV as a chronic and episodic illness. This includes examining the impact of living with multiple health conditions and their interactions, and how the health-related consequences of these conditions are experienced over time as people age with HIV. Three priorities were identified in this content area.

### Research priority 1 - episodic health and disability

Research that seeks to understand the experience of HIV as an episodic illness should consider a broad range of disability dimensions including the physical, cognitive, and mental health consequences of HIV. HIV and rehabilitation research also should focus on social participation including issues related to labor force participation, social isolation, food security, housing and poverty. Uncertainty was a dimension of disability highlighted as a priority for those aging with HIV. Uncertainty included worrying about when an episode of illness might arise and its consequences; and the long-term financial and health concerns of living with HIV. A distinct conceptualization of ‘time’ was connected with uncertainty – specifically, for people living with chronic and episodic illnesses, where there may be an altered sense of time (e.g., how long an individual perceives he/she will live, and with what quality of life, and the value of professional development, employment or relationships in the face of uncertainty). Future research should explore the complex interaction between uncertainty and time, the experience of uncertainty and clinical tools to address uncertainty about the future for people living with HIV.

A second theme was exploring the episodic nature of disability and health over time. Research should seek to identify the dimensions of disability that are episodic in nature, specifically the types of disability that fluctuate daily, within the day, and the larger fluctuations in health over the course of living with HIV. Knowing which dimensions of disability are episodic (e.g. uncertainty, physical, cognitive, mental health impairments) can help providers specifically target interventions to help mitigate the severity and frequency of episodes of illness over time (Figure 1).

### Research priority 2 - aging with HIV across the life course

Participants articulated the need for continued research on the experience of aging with HIV. Although the Forum discussion largely focused on older adults living with HIV, participants also identified a need to consider research related to children, youth and young adults living with HIV. Participants raised the need to explore factors that contribute to ‘successful’ or ‘healthy’ aging with HIV. This includes topics of how to manage stigma, resiliency, sexual health and self-efficacy. Participants also described the need to explore specific dimensions of disability with older adults with HIV. For example, research on social participation should include issues of social engagement, social isolation, mental health and well-being and the complexities of aging and employment and how retirement and financial security impact labor force participation for older adults with HIV (Figure 1).

### Research priority 3 – concurrent health conditions

Participants highlighted the need to examine the health-related consequences of concurrent health conditions experienced by people with HIV. Examples include mental health (anxiety, depression), HIV-associated neurocognitive disorder (HAND), cardiovascular disease, diabetes, bone and joint disorders, and frailty. Participants specifically emphasized the need to explore early predictors of asymptomatic neurological impairment (ANI) and minor neurocognitive disorder (MND), consider the functional and social implications of HAND, and determine the link between common concurrent health conditions (e.g. HAND and cardiovascular disease). Conditions that may not be specific to aging but may be observed in conjunction with HIV were highlighted as important to consider in future research (e.g. Hepatitis C, addictions). Understanding the complexity of disability based on the number and type of conditions can help to inform future rehabilitation in order to prevent or mitigate disability associated with HIV and concurrent conditions across the life course (Figure 1).

### Content area 2 - rehabilitation

Forum discussion focused on rehabilitation interventions and service delivery. Two research priorities were identified in this content area.

### Research priority 4 - access to rehabilitation and models of rehabilitation service provision

At times, people with HIV have to navigate through fragmented and complex systems in a climate of funding restraints and reduction of services. Participants acknowledged pressures to consider HIV within a broader chronic illness context, where disease specific services are not always accessible.

Participants highlighted the need to explore facilitators and barriers to accessing formal and informal rehabilitation services for people living with HIV. These include issues around stigma, geographical limitations (urban versus rural), gender, ethnicity, or policy barriers that influence access to rehabilitation. A need exists to increase awareness and recognition of programs and policies among clinicians and programming staff at community organizations to promote greater access to rehabilitation for people with HIV.

Participants underlined the need to develop and evaluate innovative models of rehabilitation health service delivery addressing cost effectiveness, sustainability and health outcomes. Specific recommendations included, early screening and assessment for disability to identify the need for rehabilitation, understanding the transition throughout the HIV continuum of care and, tailoring service delivery to increase the accessibility of rehabilitation to different populations (Figure 1).

### Research priority 5 - effectiveness of rehabilitation interventions

Participants identified the need to focus on evaluating the effectiveness and sustainability of rehabilitation interventions in order to mitigate the dimensions of disability experienced by people living with HIV. Specific recommendations included evaluating the effectiveness of exercise on physical, neurocognitive and mental health outcomes and determining the impact of neurocognitive (or brain) rehabilitation interventions on the overall health of people living with HIV. Mental health interventions were important to consider for their ability to reduce depression and isolation. Interventions specific to labor force participation and vocational rehabilitation emerged as an ongoing priority. Participants emphasized the need to explore predictors of returning or staying at work, interventions aimed to enhance labor force participation, and the impact of sustained employment on physical, cognitive, emotional and social health. Another focus included the need to evaluate strategies that may help to reduce the uncertainty of living with HIV. Other outcomes of interest to consider when evaluating rehabilitation interventions included medication adherence, smoking cessation, and the overall impact of rehabilitation on reducing the episodic nature of HIV-related disability. Evaluating the effectiveness of rehabilitation interventions for people living with HIV will provide a foundation for the future development of evidence-informed recommendations that will help to translate evidence to the community and overall help to guide HIV clinical practice, programming and policy (Figure 1).

### Content area 3 - methodological advances

Forum participants consistently identified the importance of research that could positively impact the lives of people living with HIV. This included evaluating interventions through the use of shared measurement tools, using recruitment strategies that would facilitate engagement of diverse populations in HIV research, and adopting innovative knowledge translation and exchange strategies with the community. Participants highlighted the importance of including individuals living with multi-morbidity in research to address the increasing complexity they see in their clients. Discussion on methodological advances focused in particular on outcome measurement tools.

### Research priority 6 - enhancing outcome measurement in hiv and rehabilitation clinical practice and research

Participants highlighted the need to enhance the use and development of patient centred outcomes in HIV, disability and rehabilitation research to facilitate communication among clinicians and evaluate the effectiveness of various interventions. Specific recommendations included choosing culturally appropriate, sensitive, valid and reliable outcome measures for use in clinical practice and research. Establishing measurement properties of newly developed HIV-specific health status instruments will be critical to provide a rigorous foundation for future research. This may include assessing the validity of instruments in varying contexts to promote universal use with people living with HIV. A need remains for the development and validation of new culturally appropriate and discriminative outcome measures in the field of HIV, disability and rehabilitation, such as screening tools better able to distinguish between milder forms of HAND, and to better determine the impact of neurocognitive impairment on the daily function and lives of people with HIV. Participants also expressed the need to establish measures that assess the episodic nature of disability over time. Finally, participants raised consideration of developing a compilation of internationally applicable patient reported outcome measures for use in HIV and rehabilitation research. This may help to facilitate cross-cultural research collaborations and enhance communication among different HIV populations internationally (Figure 1).

### Methodological considerations

While the previously described research priorities provide content-related recommendations for research, participants identified a number of methodological considerations for the process of addressing the above research priorities in HIV, disability and rehabilitation.

Participants recommended that researchers engage in both qualitative and quantitative methodological approaches as they may apply to a given research question, and include diverse, harder-to-reach, or marginalized populations in research. Researchers should consider collaborating with other disciplines such as gerontology that can lend expertise to the HIV and rehabilitation research agenda. Researchers should determine whether a research question is best addressed taking an HIV-specific or a broader episodic illness approach which would include other illness populations in a given research inquiry. These strategies may require the development of new partnerships with areas traditionally not familiar to the HIV context but of growing importance, such as the field of gerontology.

Other methodological considerations included adopting a community-engaged research approach to acknowledge the vital role that people living with HIV and the broader community have in HIV, disability and rehabilitation research. Community-engaged research also may help to raise the awareness about the roles for rehabilitation and promote the integration of rehabilitation into practice. This approach also ensures that research is meaningful to communities and promotes integrated knowledge translation with stakeholders to inform future rehabilitation practice and policy.

Participants raised the importance of considering the social determinants of health and the complex and intersecting concurrent health conditions people may experience in combination with HIV. Participants expressed concern that certain types of individuals living with complex and multiple morbidities are often excluded from studies, making research findings less representative of the population they see in their practice. Finally, researchers should consider the possibility of international research collaborations involving countries where people living with HIV may experience similar issues related to HIV, disability and rehabilitation. Future research will need to consider the benefits of taking an international approach, implications for funding and the broader applicability of results to other international contexts (Figure 1).

### Contextual factors

Forum participants highlighted the importance of considering the personal (intrinsic) or environmental (extrinsic) contextual factors (or lenses) in HIV, disability and rehabilitation research. Non-modifiable personal factors included age, ethnocultural background, and concurrent health conditions. Participants specifically highlighted the need to consider gender and ethnocultural differences aging with HIV, and the experience living with different combinations of health conditions. Environmental factors, which were considered modifiable, included considerations of geographic region (urban versus rural), stigma, policy, poverty, housing and social justice issues in HIV and rehabilitation research.

### Knowledge translation and exchange - translating research into practice, programs, and policy

Connecting research and practice emerged as a consistent theme throughout the Forum. Participants highlighted the need for research to be driven by the needs of the community and clinical practice and ensuring that research evidence is translated into appropriate programs and policy. This final component of the Framework includes recommendations aimed at maximizing the relevance and impact of research on practice, policy and programs.

Participants highlighted the importance of developing evidence-informed recommendations to translate research into practice and optimize health outcomes for people living with HIV. Knowledge translation strategies are needed to facilitate the application of research knowledge generated from these priority areas. Finally, the Forum discussion highlighted the importance of using translation of research to inform programming and policy, particularly in the current fiscal environment where rehabilitation services are sparse and fraught with barriers to access. Recommendations included considering opportunities for social media as a means for knowledge translation.

## Discussion

The Framework of Research Priorities in HIV, Disability and Rehabilitation emerged from the perspectives of researchers, clinicians, people living with HIV, representatives from community-based organizations, funders and policy stakeholders in the field of HIV and rehabilitation through the first International Forum on HIV and Rehabilitation Research. Many of the priorities overlap suggesting a given research study may collectively address a number of priorities. This Framework reflects the current and emerging priorities in the field, directly building on the original six research priorities established by CWGHR in the 2008 national scoping study [23].

Priority 1 (episodic health and disability) replaced the original ‘disability and episodic disability’ priority in the earlier scoping study. Incorporating health into the priority reflected the transition of HIV into a chronic disease, and the need to adopt a more health-oriented approach and promote self-management strategies. Evidence has explored the experience of episodic disability and relationships between dimensions of disability [10],[42], however the episodic nature of disability has not been examined longitudinally. Evidence on the barriers and facilitators to employment, and importance of employment as it relates to physical and mental health, has articulated the need for rehabilitation interventions, programs and policies to enhance the ability to recruit and retain people living with HIV in the employment sector [43]-[46]. Priorities related to episodic disability may be similarly experienced by individuals living with other chronic and episodic illnesses such as mental health, arthritis, multiple sclerosis, and some forms of cancer. The CWGHR recently launched a business case for retaining and recruiting people with episodic disabilities in the workforce, which highlights the potential for cross-disability research on employment and labor force participation [47].

Priority 2 (aging with HIV across the life course) and Priority 3 (concurrent health conditions) were originally one collective priority. Given not all comorbidities are associated with older age, we felt it was important to make them distinct in the new Framework. Emerging evidence has provided further understanding of the experience of aging with HIV, particularly highlighting the influence of stigma, and the role of uncertainty among older adults aging with HIV [48]-[50]. Uncertainty is a key component of disability, particularly for older adults living with HIV, who may worry about their source of health challenges; health providers’ knowledge and skills; financial uncertainty; transition to retirement; appropriate long-term housing and who will care for them. Evidence supports the need to focus on interventions to promote successful aging for older adults with HIV in areas including cognitive, mental and social health, productivity, personal control and life satisfaction [50],[51]. This priority will build on earlier work of resilience, optimism and mastery among older adults to explore elements of strategies for successful aging with HIV [52],[53]. Ongoing updates on evidence-informed recommendations on rehabilitation for older adults with HIV will provide guidance for clinicians working in HIV care [54].

Neurocognitive health, which may or may not be related to aging, was a focus of research priority 3. Neurocognitive impairment in the context of HIV remains prevalent hence, appropriate screening and treatment are critical [55],[56]. Standards for enhancing overall psychosocial support provide guidelines to enhance mental, cognitive, emotional and behavioral well-being for adults with HIV [57]. However, cognitive rehabilitation comprises a small component of these standards. A paucity of evidence exists on the effect of neurocognitive rehabilitation interventions for people living with HIV. Interventions should be tailored to yield ‘real world’ benefits targeted towards daily function and quality of life of people with HIV [58].

Mental health, specifically depression, was another focus of research priority 3. With persistent high rates of depression among people living with HIV [59], and its associated risk with non-adherence and decreased health-related quality of life [60],[61], screening, and establishing effective interventions for depression remains critical to future HIV clinical practice and research [62].

Priorities 2 and 3 may be considered analogous to priorities on HIV and aging established by a working group in the National Institutes of Health Office of AIDS Research [6]. These priorities similarly addressed multi-morbidity and the need to emphasize maintenance of function, and the complexity (or uncertainty) of assessing effects of HIV, treatment, and aging versus concurrent disease. The collective process of aging and the presence of concurrent health conditions are associated with functional status impairment and subsequently a determinant of frailty, a condition becoming increasingly important to consider among adults aging with HIV [63].

In this Framework, Priority 4 (rehabilitation service provision) and Priority 5 (effectiveness of rehabilitation interventions) are considered distinct; whereas they were grouped together in the earlier iteration of the priorities, highlighting the emerging evidence in these two fields.

While formalized HIV-specialist physical therapy and occupational therapy services exist in within the UK hospital environment [64], few people living with HIV access formalized rehabilitation services in Canada [17]. With the rising prevalence of chronic diseases, the Canadian Academy of Health Sciences launched a vision where people with chronic conditions should have access to health care services and clinicians who are able to recognize their needs and help address their health challenges accordingly [65]. Rehabilitation is a key component in the care continuum that may be considered situated within the HIV treatment cascade at the stage of linking to appropriate health services and support [66]. However, lack of awareness of the role for rehabilitation and the paucity of evidence on its effectiveness remain barriers to those accessing rehabilitation services. CWGHR continues to coordinate a national Equitable Access to Rehabilitation agenda[67]. With increasing rates of chronic disease among an aging HIV population, managing the complexity of episodic illnesses will make rehabilitation services even more important. Flexible person-centred care to recognize the complex and changing needs of people with HIV and episodic disability is critical for rehabilitation. Future research should explore the development and evaluation of complex integrative rehabilitation care delivery through specialty Day Health Programs, Community Health Centres, Hospitals, and AIDS Service Organizations.

Opportunities exist to explore innovative models in which to deliver rehabilitation care, interventions and support. Self-management has become increasingly important now that HIV is recognized as a chronic illness, associated with improvements in symptom management, anxiety, and medication adherence for PHAs [68]-[71]. Self-management interventions in the context of HIV can include self-care, interpersonal skills, technical knowledge, cognitive skills, positive attitudes, planning for the future, and role management [72]. Exercise is one self-management living strategy that may be adopted by adults with HIV to prevent disability and enhance health. Systematic reviews suggest exercise is safe and may lead to benefits in cardiopulmonary fitness, strength, weight and body composition, and psychological status for people with HIV [73],[74]. Despite these known benefits, few PHAs engage in exercise [75]. Authors have described factors that influence adherence to exercise (and other interventions) in people living with HIV [76],[77]. Forum discussion highlighted the importance to consider adherence over the long-term in future intervention research as this is critical for adopting healthy living strategies.

Priority 6 highlights the importance of self-reported health outcomes in measuring the health-related consequences of HIV, aging and comorbidities and effectiveness of interventions. Physical and psychosocial dimensions of disability are associated with self-reported health status and important to consider in HIV care [78]. The Assessment of Motor Performance Scale (AMPS) [79], HIV Disability Questionnaire (HDQ) [80],[81], and measures of frailty [82] were examples of outcomes used in practice and research discussed at the Forum. Consideration of disability outcomes are important to consider in HIV research. Outcomes that assess disability may be considered part of a universal pool of agreed upon measures in HIV, disability and rehabilitation research. The need to utilize well-validated indices in HIV human research has similarly been articulated in the context of HIV and aging [6].

Knowledge translation is an integral component of the Framework. It is critical researchers consider integrated knowledge transfer and exchange of evidence. Implementation science has become increasingly important to consider methods to promote the integration of research findings into HIV health care policy and practice [83]. Stakeholders should consider mechanisms in which to ensure research evidence is translated to inform practice, program and policy in order to enhance health outcomes of people with HIV. Examples include strategies to increase knowledge about HIV care among rehabilitation professionals through the uptake of an electronic module on HIV rehabilitation [84] or conducting a knowledge synthesis to develop evidence-informed recommendations on rehabilitation for clinicians working in HIV care [54]. Community-based research can also help to enhance knowledge translation and exchange with community members and organizations to ensure research is relevant, and so that it can more effectively move into practice [85]. Finally, the nature of our Forum approach, characterized by international, multi-sectoral and interdisciplinary stakeholder engagement ensured broad translation of research on HIV, disability and rehabilitation. Development of a Knowledge Translation and Exchange (KTE) Library comprised of speaker slides, research evidence panel films, and rapporteur notes further broadened the uptake of the evidence presented at the Forum [37]. Overall, the knowledge translation, methodological considerations, and contextual factor components of the Framework are intended to guide the research process and its translation into programs and policy.

### Strengths and Limitations

Our approach establishing new and emerging research priorities involved a multi-stakeholder consultation at the International Forum on HIV and Rehabilitation Research. We engaged a broad range of international stakeholders, with expertise in issues related to HIV and rehabilitation. Second, this Framework builds on the foundational work by CWGHR who established the original six priorities in 2008. [23] This enabled stakeholders to reflect and consider changes related to HIV knowledge and treatment, shifts in the demographic profile of populations living with HIV, and changes related to health care systems. The Framework of Research Priorities in HIV, Disability and Rehabilitation reflects the changing tide of the HIV environment as it relates to the increasing complexities of multi-morbidity in HIV and the changing health system environment, influencing access to rehabilitation care. The Framework goes beyond the medical model, focused on virological or immunological outcomes of health, to emphasize the need to consider the consequences of disease (disability) and the role for rehabilitation in addressing disability. Third, the priorities in the Framework are evidence-based, building on the foundational research that was presented at the Forum. The priorities propose further critical inquiry and examination in order to promote timely and effective rehabilitation interventions and impact policy and practice. Fourth, these priorities emerged from five different mechanisms of consultation with various stakeholders involved in the Forum. Finally, the process in which the priorities were derived involved multiple perspectives and was refined with the research priority Forum working group.

Our approach was not without limitations. Although Forum participants were asked to broadly consider new and emerging issues related to HIV and rehabilitation, discussion and feedback was framed by the panel sessions which were based on the original six research priorities developed by CWGHR. Given we built on the original priorities, we did not conduct a second scoping study of HIV and rehabilitation research. However, the vast expertise in HIV and rehabilitation among participants at the Forum which focused on knowledge translation increased the likelihood that any newly published evidence since the original scoping study were likely discussed at the Forum. The research priorities were derived largely from the Canadian and UK perspective. Hence, the applicability of the Framework to other countries including the developing context is unknown. Future International Forums may consider expanding representation from other countries where the role for rehabilitation is emerging. Lastly, the Framework provides recommendations for broad content areas in which to pursue in HIV, disability and rehabilitation research. Next steps will be for researchers and clinicians to develop specific research questions and methodologies derived from these key priorities.

## Conclusions

The need for research in the field of HIV, disability and rehabilitation continues to rise. This paper presents a Framework of Research Priorities in HIV, Disability and Rehabilitation comprised of six priority areas: episodic health and disability, concurrent health conditions, aging with HIV across the life course, access to rehabilitation and models of rehabilitation service provision, effectiveness of rehabilitation interventions, and enhancing outcome measurement. These priorities outline a future plan for HIV, disability and rehabilitation research that will help increase our knowledge to enhance practice, programming and policy for people living with HIV.

## Authors’ original submitted files for images

Below are the links to the authors’ original submitted files for images. Authors’ original file for figure 1

## References

[1] Samji H, Cescon A, Hogg RS, Modur SP, Althoff KN, Buchacz K, Burchell AN, Cohen M, Gebo KA, Gill MJ, Justice A, Kirk G, Klein MB, Korthuis T, Martin J, Napravnik S, Rourke SB, Sterling TR, Silverberg MJ, Deeks S, Jacobson LP, Bosch RJ, Kitahata MM, Goedert JJ, Moore R, Gange SJ: for The North American AIDS Cohort Collaboration on Research and Design (NA-ACCORD) of IeDEA: Closing the gap: increases in life expectancy among treated HIV-positive individuals in the United States and Canada. *PLoS One* 2013, 8:e81355., 10.1371/journal.pone.0081355 PMC3867319 24367482

[2] Public Health Agency of Canada (2011). Summary: Estimates of HIV Prevalence and Incidence in Canada.

[3] Centers for Disease Control (2013). Monitoring selected national HIV prevention and care objectives by using HIV surveillance data - United States and 6 U.S. dependent areas—2011. HIV Surveillance Supplemental Report.

[4] Public Health England (2013). HIV in the United Kingdom: 2013 report.

[5] Deeks SG, Lewin SR, Havlir DV (2013). The end of AIDS: HIV infection as a chronic disease. Lancet.

[6] High KP, Brennan-Ing M, Clifford DB, Cohen MH, Currier J, Deeks SG, Deren S, Effros RB, Gebo K, Goronzy JJ, Justice AC, Landay A, Levin J, Miotti PG, Munk RJ, Nass H, Rinaldo CR, Shlipak MG, Tracy R, Valcour V, Vance DE, Walston JD, Volberding P, OAR Working Group on HIV and Aging (2012). HIV and aging: state of knowledge and areas of critical need for research. A report to the NIH Office of AIDS Research by the HIV and Aging Working Group. J Acquir Immune Defic Syndr.

[7] Palella FJ, Baker RK, Moorman AC, Chmiel JS, Wood KC, Brooks JT, Holmberg SD (2006). Mortality in the highly active antiretroviral therapy era: changing causes of death and disease in the HIV outpatient study. J Acquir Immune Defic Syndr.

[8] Rusch M, Nixon S, Schilder A, Braitstein P, Chan K, Hogg RS: Impairments, activity limitations and participation restrictions: prevalence and associations among persons living with HIV/AIDS in British Columbia. *Health Qual Life Outcomes* 2004, 2:46., 10.1186/1477-7525-2-46 PMC519026 15350202

[9] Willard S, Holzemer WL, Wantland DJ, Cuca YP, Kirksey KM, Portillo CJ, Corless IB, Rivero-Mendez M, Rosa ME, Nicholas PK, Hamilton MJ, Sefcik E, Kemppainen J, Canaval G, Robinson L, Moezzi S, Human S, Arudo J, Eller LS, Bunch E, Dole PJ, Coleman C, Nokes K, Reynolds NR, Tsai YF, Maryland M, Voss J, Lindgren T (2009). Does “asymptomatic” mean without symptoms for those living with HIV infection?. AIDS Care.

[10] O'Brien KK, Bayoumi AM, Strike C, Young NL, Davis AM: Exploring disability from the perspective of adults living with HIV/AIDS: development of a conceptual framework. *Health Qual Life Outcomes* 2008, 6:76., 10.1186/1477-7525-6-76 PMC2572592 18834538

[11] O'Brien KK, Davis AM, Strike C, Young NL, Bayoumi AM: Putting episodic disability into context: a qualitative study exploring factors that influence disability experienced by adults living with HIV/AIDS. *J Int AIDS Soc* 2009, 12:5., 10.1186/1758-2652-12-30 PMC2788343 19900284

[12] Worthington C, Myers T, O'Brien K, Nixon S, Cockerill R (2005). Rehabilitation in HIV/AIDS: development of an expanded conceptual framework. AIDS Patient Care STDS.

[13] Fish G, Rudman DL (1998). The potential role of occupational therapy in acute care with clients with HIV/AIDS. Occup Ther Int.

[14] Nixon S, Cott C (2000). Shifting perspectives: reconceptualizing HIV disease within a rehabilitation framework. Physiotherapy Canada.. Physiother Can.

[15] Lewis DL, Abernathy T, Molloy DW, Connelly D, Knott TC, Mngoma N, Coulas G, Breau R (2006). Demand for rehabilitation of Ontario’s elderly: a social forecasting approach.

[16] Landry MD, Jaglal S, Wodchis WP, Raman J, Cott CA (2008). Analysis of factors affecting demand for rehabilitation services in Ontario, Canada. A health policy perspective. Disabil Rehabil.

[17] Worthington C, Myers T, O'Brien K, Nixon S, Cockerill R, Bereket T (2008). Rehabilitation professionals and human immunodeficiency virus care: results of a national Canadian survey. Arch Phys Med Rehabil.

[18] Worthington C, O'Brien K, Myers T, Nixon S, Cockerill R (2009). Expanding the lens of HIV services provision in Canada: results of a national survey of HIV health professionals. AIDS Care.

[19] Agnoletto V, Chiaffarino F, Nasta P, Rossi R, Parazzini F (2006). Use of complementary and alternative medicine in HIV-infected subjects. Complement Ther Med.

[20] Crook J, Browne G, Roberts J, Gafni A (2005). Impact of support services provided by a community-based AIDS service organization on persons living with HIV/AIDS. J Assoc Nurses AIDS Care.

[21] Furler MD, Einarson TR, Walmsley S, Millson M, Bendayan R (2003). Use of complementary and alternative medicine by HIV-infected outpatients in Ontario, Canada. AIDS Patient Care STDS.

[22] Littlewood RA, Vanable PA (2008). Complementary and alternative medicine use among HIV-positive people: research synthesis and implications for HIV care. AIDS Care.

[23] O'Brien K, Wilkins A, Zack E, Solomon P (2010). Scoping the Field: Identifying Key Research Priorities in HIV and Rehabilitation. AIDS Behav.

[24] Canadian Working Group on HIV and Rehabilitation: *HIV, disability and rehabilitation: Promoting quality of life through research, education and cross-sector partnerships. Strategic Plan* 2010–2013. 2010.

[25] Vance DE, Mugavero M, Willig J, Raper JL, Saag MS (2011). Aging With HIV: A Cross-Sectional Study of Comorbidity Prevalence and Clinical Characteristics Across Decades of Life. J Assoc Nurses AIDS Care.

[26] Balogun JA, Kaplan MT, Miller TM (1998). The effect of professional education on the knowledge and attitudes of physical therapist and occupational therapist students about acquired immunodeficiency syndrome. Phys Ther.

[27] Brown TT, Qaqish RB (2006). Antiretroviral therapy and the prevalence of osteopenia and osteoporosis: a meta-analytic review. AIDS.

[28] Heaton RK, Clifford DB, Franklin DR, Woods SP, Ake C, Vaida F, Ellis RJ, Letendre SL, Marcotte TD, Atkinson JH, Rivera-Mindt M, Vigil OR, Taylor MJ, Collier AC, Marra CM, Gelman BBB, McArthur JC, Morgello S, Simpson DM, McCutchan JA, Abramson I, Gamst A, Fennema-Notestine C, Jernigan TL, Wong J, Grant I (2011). CHARTER Group: **HIV**-**associated neurocognitive disorders persist in the era of potent antiretroviral therapy**: **CHARTER Study**. Neurology.

[29] Shiels MS, Cole SR, Kirk GD, Poole C (2009). A meta-analysis of the incidence of non-AIDS cancers in HIV-infected individuals. J Acquir Immune Defic Syndr.

[30] Kendall CE, Wong J, Taljaard M, Glazier RH, Hogg W, Younger J, Manuel DG: A cross-sectional, population-based study measuring comorbidity among people living with HIV in Ontario. *BMC Publ Health* 2014, 14:161., 10.1186/1471-2458-14-161 PMC3933292 24524286

[31] Hasse B, Ledergerber B, Furrer H, Battegay M, Hirschel B, Cavassini M, Bertisch B, Bernasconi E, Weber R (2011). Morbidity and aging in HIV-infected persons: the Swiss HIV cohort study. Clin Infect Dis.

[32] Guaraldi G, Orlando G, Zona S, Menozzi M, Carli F, Garlassi E, Berti A, Rossi E, Roverato A, Palella F (2011). Premature age-related comorbidities among HIV-infected persons compared with the general population. Clin Infect Dis.

[33] Valcour V, Shikuma C, Shiramizu B, Watters M, Poff P, Selnes O, Holck P, Grove J, Sacktor N (2004). Higher frequency of dementia in older HIV-1 individuals: the Hawaii Aging with HIV-1 Cohort. Neurology.

[34] Havlik RJ, Brennan M, Karpiak SE (2011). Comorbidities and depression in older adults with HIV. Sex Health.

[35] Shippy RA, Karpiak SE (2005). The aging HIV/AIDS population: fragile social networks. Aging Ment Health.

[36] Roger KS, Mignone J, Kirkland S (2013). Social aspects of HIV/AIDS and aging: a thematic review. Can J Aging.

[37] Canada-United Kingdom HIV and Rehabilitation Research: Collaborative (CUHRRC): *International Forum on HIV and Rehabilitation Research.* June 2013. [http://www.hivandrehab.ca/EN/AGM2013/]

[38] Canada-United Kingdom HIV and Rehabilitation Research: Collaborative (CUHRRC): *International Forum on HIV and Rehabilitation Research: Translating Research Evidence from the Canada-UK HIV and Rehabilitation Research Collaborative (CUHRRC) and Canadian Working Group on HIV and Rehabilitation Research Collaborative (CWGHR).* 2013 [http://www.hivandrehab.ca/EN/AGM2013/documents/Forum-Program-Workbook-FINAL-CLEAN-June-7-13ENGLISH.pdf].

[39] Hsieh HF, Shannon SE (2005). Three approaches to qualitative content analysis. Qual Health Res.

[40] Sackett DL, Rosenberg WM, Gray JA, Haynes RB, Richardson WS (1996). Evidence based medicine: what it is and what it isn't. BMJ.

[41] Straus SE, Richardson WE, Glasziou P, Haynes RB (2011). Evidence-Based Medicine: How to Practice and Teach it.

[42] O'Brien KK, Davis AM, Gardner S, Bayoumi AM, Rueda S, Hart TA, Cooper C, Solomon P, Rourke SB, Hanna S (2014). Relationships Between Dimensions of Disability Experienced by Adults Living with HIV: A Structural Equation Model Analysis. AIDS Behav.

[43] Worthington C, O'Brien K, Zack E, McKee E, Oliver B (2012). Enhancing Labour Force Participation for People Living with HIV: A Multi-Perspective Summary of the Research Evidence. AIDS Behav.

[44] Rueda S, Chambers L, Wilson M, Mustard C, Rourke SB, Bayoumi A, Raboud J, Lavis J (2012). Association of returning to work with better health in working-aged adults: a systematic review. Am J Public Health.

[45] Rueda S, Raboud J, Plankey M, Ostrow D, Mustard C, Rourke SB, Jacobson LP, Bekele T, Bayoumi A, Lavis J, Detels R, Silvestre AJ (2012). Labor force participation and health-related quality of life in HIV-positive men who have sex with men: the Multicenter AIDS Cohort Study. AIDS Behav.

[46] Rueda S, Raboud J, Rourke SB, Bekele T, Bayoumi A, Lavis J, Cairney J, Mustard C (2012). Influence of employment and job security on physical and mental health in adults living with HIV: cross-sectional analysis. Open Med.

[47] Canadian Working Group on HIV and Rehabilitation: *A Win-Win Proposition: The Business Case for Actively Recruiting and Retaining People with Episodic Disabilities.* 2014.

[48] Emlet CA, Brennan DJ, Brennenstuhl S, Rueda S, Hart TA, Rourke SB (2013). Protective and risk factors associated with stigma in a population of older adults living with HIV in Ontario, Canada. AIDS Care.

[49] Emlet CA (2007). Experiences of stigma in older adults living with HIV/AIDS: a mixed-methods analysis. AIDS Patient Care STDS.

[50] Solomon P, O'Brien K, Wilkins S, Gervais N (2014). Aging with HIV and disability: the role of uncertainty. AIDS Care.

[51] Vance DE, McGuinness T, Musgrove K, Orel NA, Fazeli PL (2011). Successful aging and the epidemiology of HIV. Clin Interv Aging.

[52] Emlet CA, Tozay S, Raveis VH (2011). “I'm not going to die from the AIDS”: resilience in aging with HIV disease. Gerontologist.

[53] Moore RC, Moore DJ, Thompson WK, Vahia IV, Grant I, Jeste DV (2013). A case-controlled study of successful aging in older HIV-infected adults. J Clin Psychiatry.

[54] O'Brien KK, Solomon P, Trentham B, MacLachlan D, MacDermid J, Tynan AM, Baxter L, Casey A, Chegwidden W, Robinson G, Tran T, Wu J, Zack E: Evidence-informed recommendations for rehabilitation with older adults living with HIV: a knowledge synthesis. *BMJ Open* 2014, 4:e004692., 10.1136/bmjopen-2013-004692 PMC4024604 24833687

[55] Rackstraw S, Chegwidden W (2013). HIV and rehabilitation research and service delivery in the United Kingdom.

[56] Mind Exchange Working Group (2013). Assessment, diagnosis, and treatment of HIV-associated neurocognitive disorder: a consensus report of the mind exchange program. Clin Infect Dis.

[57] British Psychological Society (2011). British HIV Association & Medical Foundation for AIDS & Sexual Health: Standards for psychological support for adults living with HIV.

[58] Weber E, Blackstone K, Woods SP (2013). Cognitive neurorehabilitation of HIV-associated neurocognitive disorders: a qualitative review and call to action. Neuropsychol Rev.

[59] Valente SM (2003). Depression and HIV disease. J Assoc Nurses AIDS Care.

[60] DiMatteo MR, Lepper HS, Croghan TW (2000). Depression is a risk factor for noncompliance with medical treatment: meta-analysis of the effects of anxiety and depression on patient adherence. Arch Intern Med.

[61] Jia H, Uphold CR, Wu S, Chen GJ, Duncan PW (2005). Predictors of changes in health-related quality of life among men with HIV infection in the HAART era. AIDS Patient Care STDS.

[62] Simoni JM, Safren SA, Manhart LE, Lyda K, Grossman CI, Rao D, Mimiaga MJ, Wong FY, Catz SL, Blank MB, DiClemente R, WIlson IB (2011). Challenges in addressing depression in HIV research: assessment, cultural context, and methods. AIDS Behav.

[63] Guaraldi G, Silva AR, Stentarelli C (2014). Multimorbidity and functional status assessment. Curr Opin HIV AIDS.

[64] Brown D, Nelson M (2013). A review of referrals and interventions within a specialist HIV outpatient physiotherapy service. Abstracts of the 19th Annual Conference of the British HIV Association (BHIVA), Manchester, UK. HIV Med.

[65] Nasmith L, Ballem P, Baxter R, Bergman H, Colin-Thomé D, Herbert C, Keating N, Lessard R, Lyons R, McMurchy D, Ratner P, Rosenbaum P, Tamblyn R, Wagner E, Zimmerman B: *Transforming care for Canadians with chronic health conditions: Put people first, expect the best, manage for results.* Canadian Academy of Health Sciences; 2010.

[66] Hull MW, Wu Z, Montaner JS (2012). Optimizing the engagement of care cascade: a critical step to maximize the impact of HIV treatment as prevention. Curr Opin HIV AIDS.

[67] Canadian Working Group on HIV and Rehabilitation (CWGHR). *Equitable Access to Rehabilitation: Realizing Potential, Promising Practices, and Policy Directions. Discussion Paper.* February 2012. [http://hivandrehab.ca/EN/resources/documents/EquitableAccesstoRehabilitationDiscussionPaper-Final.pdf].

[68] Rozman DR, Whitaker T, Beckman DJ (1996). A pilot intervention program that reduces psychological symptomatology in indivdiuals wiht human immunodeficiency virus. Compl Ther Med.

[69] Gifford AL, Sengupta S (1999). Self-management health education for chronic HIV infection. AIDS Care.

[70] Kemppainen JK, Eller LS, Bunch E, Hamilton MJ, Dole P, Holzemer W, Kirksey K, Nicholas PK, Corless IB, Coleman C, Nokes KM, Reynolds N, Sefcik L, Wantland D, Tsai YF (2006). Strategies for self-management of HIV-related anxiety. AIDS Care.

[71] Smith SR, Rublein JC, Marcus C, Brock TP, Chesney MA (2003). A medication self-management program to improve adherence to HIV therapy regimens. Patient Educ Counsel.

[72] Bernardin KN, Toews DN, Restall GJ, Vuongphan L (2013). Self-management interventions for people living with human immunodeficiency virus: a scoping review. Can J Occup Ther.

[73] O'Brien K, Nixon S, Tynan AM, Glazier R: Aerobic exercise interventions for adults living with HIV/AIDS. *Cochrane Database Syst Rev* 2010, 8:CD001796., 10.1002/14651858.CD001796.pub3 PMC7061352 20687068

[74] O'Brien K, Tynan AM, Nixon S, Glazier RH (2008). Effects of progressive resistive exercise in adults living with HIV/AIDS: systematic review and meta-analysis of randomized trials. AIDS Care.

[75] Fillipas S, Bowtell-Harris CA, Oldmeadow LB, Cicuttini F, Holland AE, Cherry CL (2008). Physical activity uptake in patients with HIV: who does how much?. Int J STD AIDS.

[76] Petroczi A, Hawkins K, Jones G, Naughton DP (2010). HIV Patient Characteristics that Affect Adherence to Exercise Programmes: An Observational Study. Open AIDS J.

[77] Jones G, Hawkins K, Mullin R, Nepusz T, Naughton DP, Sheeran P, Petroczi A: Understanding how adherence goals promote adherence behaviours: a repeated measure observational study with HIV seropositive patients. *BMC Publ Health* 2012, 12:587., 10.1186/1471-2458-12-587 PMC3490813 22853824

[78] Harding R, Clucas C, Lampe FC, Date HL, Fisher M, Johnson M, Edwards S, Anderson J, Sherr L (2012). What factors are associated with patient self-reported health status among HIV outpatients? A multi-centre UK study of biomedical and psychosocial factors. AIDS Care.

[79] Merritt B, Gahagan J, Kottorp A: HIV and disability: a pilot study exploring the use of the Assessment of Motor and Process Skills to measure daily life performance. *J Int AIDS Soc* 2013, 16:17339., 10.7448/IAS.16.1.17339 PMC3551982 23336724

[80] O’Brien KK, Bayoumi AM, King K, Alexander R, Solomon P: Community Engagement in Health Status Instrument Development: Experience with the HIV Disability Questionnaire. *Progress in Community Health Partnerships: Research, Education and Action* In Press., 10.1353/cpr.2014.0071 25727988

[81] O'Brien KK, Bayoumi AM, Bereket T, Swinton M, Alexander R, King K, Solomon P (2013). Sensibility assessment of the HIV Disability Questionnaire. Disabil Rehabil.

[82] Fried LP, Tangen CM, Walston J, Newman AB, Hirsch C, Gottdiener J, Seeman T, Tracy R, Kop WJ, Burke G, McBurnie MA (2001). Frailty in older adults: evidence for a phenotype. J Gerontol A Biol Sci Med Sci.

[83] Glasgow RE, Eckstein ET, Elzarrad MK (2013). Implementation science perspectives and opportunities for HIV/AIDS research: integrating science, practice, and policy. J Acquir Immune Defic Syndr.

[84] Solomon P, Salbach N, O’Brien KK, Worthington C, Baxter L, Blanchard G, Casey A, Chegwidden W, Dolan L, Eby S, Gervais N (2013). Increasing capacity in rehabilitation in the management of HIV: a case-based e-mail intervention.

[85] Wilson MG, Lavis JN, Travers R, Rourke SB: Community-based knowledge transfer and exchange: helping community-based organizations link research to action. *Implement Sci* 2010, 5:33., 10.1186/1748-5908-5-33 PMC2873302 20423486

