# Supplementary figures and images for: Advancing research and practice in HIV and rehabilitation: a framework of research priorities in HIV, disability and rehabilitation
Source: BMC Infect Dis. 2014 Dec 31;14:724. doi: 10.1186/s12879-014-0724-8 (PMC4304172; doi:10.1186/s12879-014-0724-8)

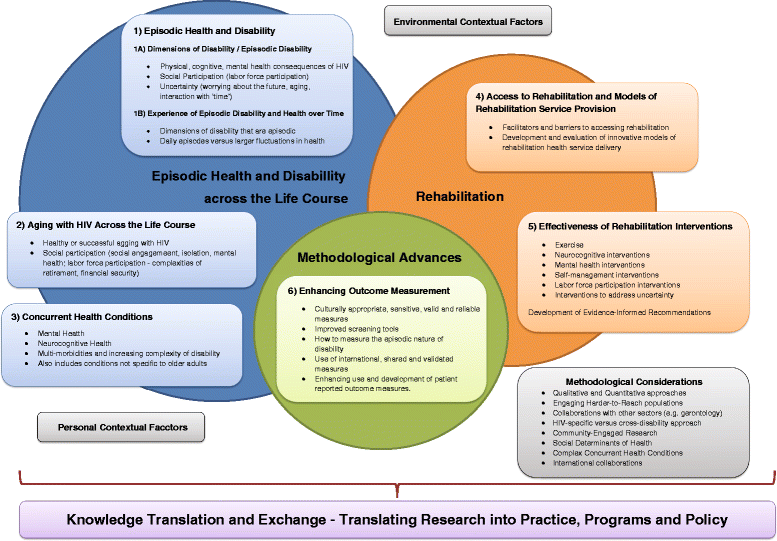

Supplement: Supplementary file 1 — Authors’ original file for figure 1 [file 12879_2014_724_MOESM1_ESM.gif]
